# Diabetes induces changes in neuroretina before retinal vessels: a spectral-domain optical coherence tomography study

**DOI:** 10.1186/s40942-015-0001-z

**Published:** 2015-04-15

**Authors:** Eduardo Büchele Rodrigues, Müller Gonçalves Urias, Fernando Marcondes Penha, Emmerson Badaró, Eduardo Novais, Rodrigo Meirelles, Michel Eid Farah

**Affiliations:** Department of Ophthalmology, Federal University of São Paulo, R. Botucatu 820, 04023-062 SP São Paulo, Brazil

**Keywords:** Diabetes mellitus, Diabetic retinopathy, OCT, Optical coherence tomography, Macula, Retina, Neuronal, Neurodegeneration

## Abstract

**Purpose:**

To investigate retinal changes prior to vascular signs in patients with type 2 diabetes without diabetic retinopathy or with mild non proliferative diabetic retinopathy.

**Methods:**

A cross-sectional study was performed in three groups: patients without diabetes, patients with type 2 diabetes without diabetic retinopathy, and patients with diabetes with mild diabetic retinopathy. Analysis of retinal layers was performed objectively with the Cirrus Review Software 6.0 (Carl Zeiss Meditec, Dublin, CA, USA). Macular cube scans were analyzed with regard to: the ganglion cell layer + inner plexiform layer analysis, retinal nerve fiber layer thickness, central subfoveal retinal thickness and average macular thickness.

**Results:**

In total, 102 patients were included in this study, of which 28 (27.4%) were classified into control group, 46 (45.0%) classified as diabetic patients with no diabetic retinopathy and 28 (27.4%) classified as mild diabetic retinopathy. Quantitative analysis with the Cirrus software showed that the mean ganglion cell layer and mean retinal nerve fiber layer were thinner in diabetes without diabetic retinopathy group when compared to controls. ANOVA with Bonferroni *post test* indicated a statistically significant reduction in average retinal thickness in mild diabetic retinopathy group (P = 0.032) compared to control and reduction in ganglion cell layer in diabetes with no diabetic retinopathy (P = 0.039) and mild diabetic retinopathy (P = 0.003). Also indicated reduction in retinal nerve fiber layer in diabetic without diabetic retinopathy and eyes with mild diabetic retinopathy (P < 0.001), compared to controls.

**Conclusions:**

Our study found reduction in thickness of ganglion cell layer and retinal nerve fiber layer in patients with diabetes without diabetic retinopathy, which suggests neuroretinal changes before vascular signs of diabetic retinopathy.

## Background

Diabetic retinopathy (DR) is considered one of the main causes of blindness in patients between 30 and 60 years old in the Western world. Despite recent progress, current treatment with pharmacologic and laser treatment may not be enough in some patients to prevent blindness. DR has been considered mostly a vascular disease, but recent investigations have demonstrated degenerative and neuronal alterations before the appearance of microvascular changes in patients with diabetes mellitus (DM) [1,2]. Animal and human studies support the presence of neuronal alterations including apoptosis in early stages of diabetes [3-6].

Optical coherence tomography (OCT) enables deep comprehension of a variety of eye diseases [7,8]. Newer generation spectral domain OCT allows detailed examination of retinal cells and vessels, thereby facilitating the study of disease pathogenesis. Clinically, different authors have reported decrease in total central retinal or single cellular layer thickness in diabetic eyes with or without clinical signs of DR compared to control groups (subjects with no DM) [1,2]. Others have shown reduction in the inner retinal thickness in the macula in diabetics with mild DR, which may represent initial ganglion cell loss in the pericentral areas followed by retinal nerve fiber layer (RNFL) thinning in the peripheral macula [5,6,9].

The goal of this study was to investigate retinal changes prior to vascular signs in patients with type 2 diabetes without diabetic retinopathy or with mild non proliferative diabetic retinopathy.

## Methods

A cross-sectional study was performed with enrollment of three groups: (A) control patients without diabetes; (B) patients with type 2 diabetes with no clinical or angiographically diagnosed DR, and (C) patients with type 2 diabetes mellitus with mild DR. Patients were examined in the Department of Ophthalmology at the Federal University of São Paulo after IRB approval, and signed informed consent to comply with voluntary participation in this research project. The institution “Comitê de Ética da Universidade Federal de São Paulo” approved this research project. Experiments were conducted according to Declaration of Helsinki.

Control subjects did not have a diagnosis of diabetes, any ocular disease, or any other systemic disease. These subjects were randomly recruited from individuals accompanying patients visiting the Department of Ophthalmology.

The inclusion criteria was individuals with type 2 DM and above the age of 40 years. The choice of type 2 diabetes was due to its prevalence and importance of future projection. Mild DR was considered as the presence of at least one microaneurysm in the retina, but no other diabetic lesions - according to the classification of the international clinical diabetic retinopathy disease severity scale [9]. Patients were excluded from the study if they presented with a best corrected visual acuity less than 20/25, when OCT images were of inadequate quality (sinal strength below 7), if DR equal or worse than moderate, lens opacity and other vision impairing diseases such as glaucoma, cataract, uveitis, or macular degeneration.

All patients underwent clinical examination with review of medical history. The following variables were collected: time of diabetes, last glucose, last glycosylated hemoglobin (HbA1c), systemic hypertension (yes or no), nephropathy (yes or no), best corrected visual acuity. Patients were evaluated with respect to best corrected visual acuity using the Snellen chart. The patients had pupil dilation by tropicamide and evaluation through digital color fundus photography, fluorescein angiography (FA) and spectral-domain optical coherence tomography (SD-OCT) (Cirrus ™ HD-OCT 4000, Carl Zeiss Meditec, Dublin, CA, USA). FA and SD-OCT were performed in the mornings.

FA examination was used as a screening to verify angiography criteria and was performed by a different examiner from the OCT images. Retina specialists who analyzed OCT images neither took part in the screening, nor they were aware details about individuals of the screening.

Only SD-OCT analysis of right eye was performed, centered on the fovea and repeated three times by the examiner. Only scans with signal strengths ≥7 and without artifact were included in the study. The acquisition protocols were as follows: macular cube 200 × 200; HD 5 line raster/5 raster line; and HD one line. The raw OCT datasets were exported to a personal computer for analysis.

Analysis of retinal layers of the right eye was performed using specific software, Cirrus HD-OCT Review Software 6.0 (Carl Zeiss Meditec, Dublin, CA, USA). The software, patented by Carl Zeiss Meditec, measured the various retinal layers in order to produce data for further analysis. Two types of measurements were performed: automated and subjective (by software caliper).

Macular cube scan generated the following automated data: ganglion cell layer + inner plexiform layer (GCL + IPL) thickness, RNFL thickness, central subfoveal retinal thickness (CS), and average retinal thickness (RT). Average thicknesses of eight areas were examined for each scan, determined by ETDRS grid: nasal, superior, temporal, inferior, nasal superior, nasal inferior, temporal superior and temporal inferior.

Line scans were performed in order to proceed the subjective analysis, only. Line scans used were centered on fovea, with a 0° angle. The subjective analysis was performed by two independent retina specialists, who have not taken part in the screening. They used Cirrus HD-OCT Review Software 6.0 (Carl Zeiss Meditec, Dublin, CA, USA) caliper and measured layers 500 μm away from the foveal center, near to optical disc. The following layers were measured: RNFL, ganglion cell layer (GCL), outer nuclear layer (ONL), inner nuclear layer (INL), outer plexiform layer (OPL), inner plexiform layer (IPL), and center foveal thickness (CS).

Analysis of variance with the Bonferroni post test was conducted. Furthermore, reproducibility of subjective measurements was analyzed, calculating the intraclass correlation coefficient (ICC). A high ICC value indicates small fluctuations between measurements from different analyzers in the same individuals, indicating a high reproducibility. However, a small ICC value shows large fluctuations, featuring a low reproducibility. The maximum value of the ICC is 1.0, while its minimum value is theoretically zero. Values higher than 0.75 were considered acceptable. Values between 0.4 and 0.75 were considered moderate and below 0.4, poor.

Data were collected and statistical analysis was performed with SPSS (version 19; SPSS, Inc., Chicago, IL, USA). Parametric and non-parametric tests were conducted using analysis of variance (ANOVA) to compare retinal changes, clinical data observed in the groups. For comparison of means, a post hoc correction for multiple testing was applied by the Bonferroni post test method. A multiple linear regression model was used to determine the relationship between inner retinal layer thickness and the duration of DM, DR status, age, sex, and HbA1c in the diabetic patients. The results were expressed as mean ± SD if the variables were continuous, and as percentage if categorical.

## Results

**Figure 1:**
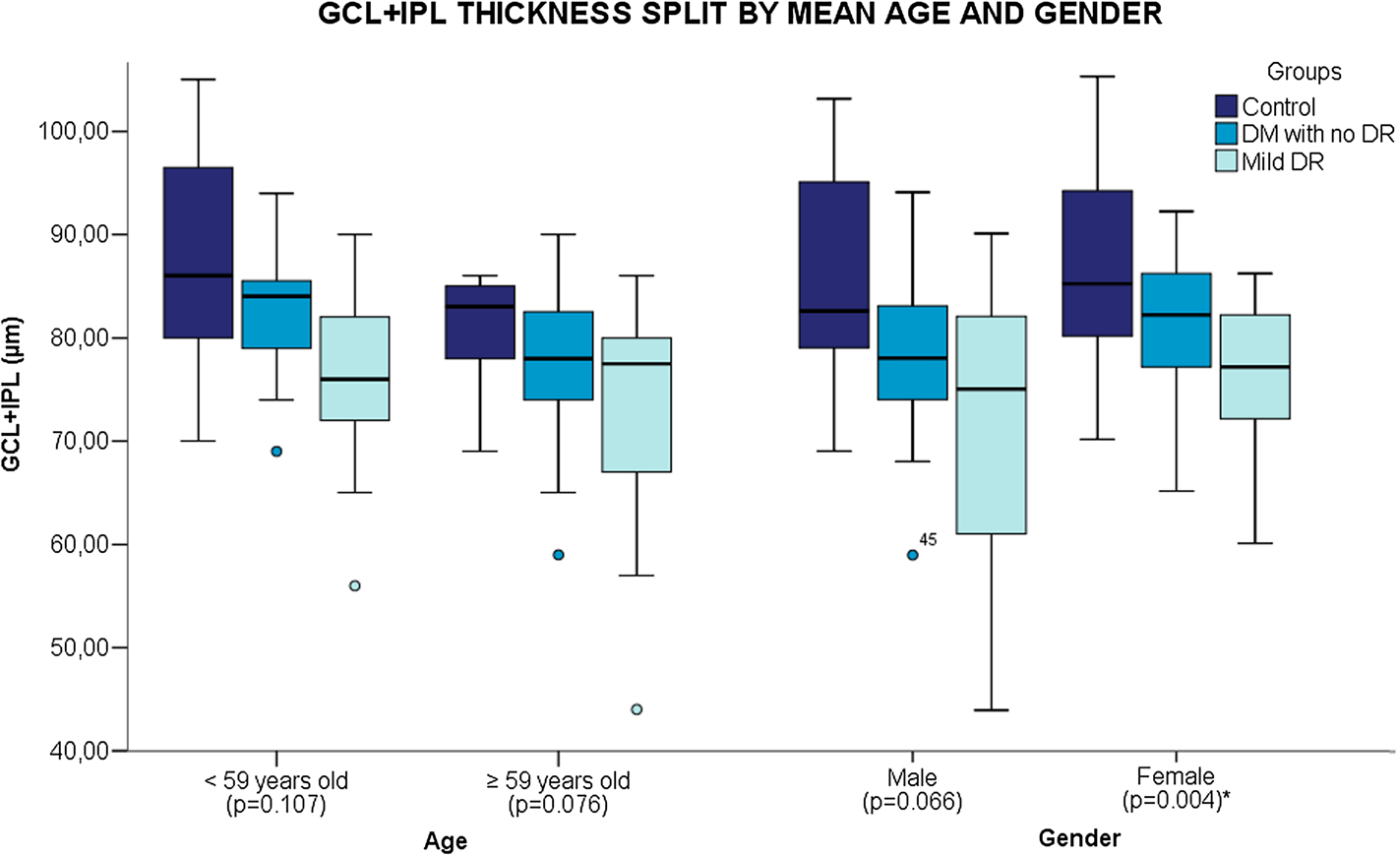
**Boxplot shows mean ganglion cell + inner plexiform layer (GC + IPL) thickness of each study group split by mean age and gender.** There was a statistically significant reduction in GCL + IPL between groups in female patients.

**Figure 2:**
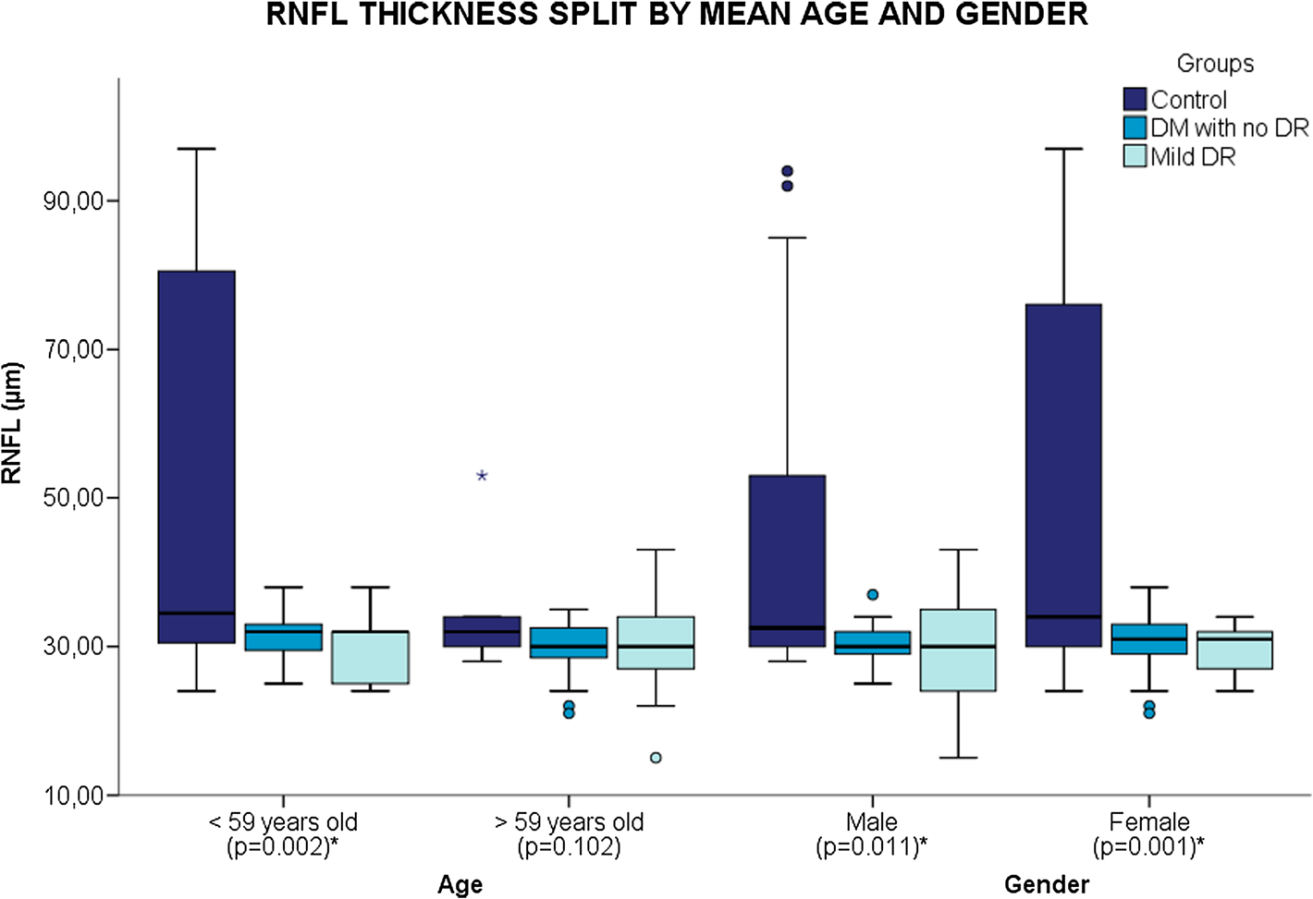
**Boxplot shows mean retinal nerve fiber layer (RNFL) thickness of each study group split by mean age and gender.** There was a statistically significant reduction in RNFL between groups in patients with age below 59 years old and both gender.

In total, 102 patients were included in this study, of which 28 (27.4%) were classified into control group, 46 (45.0%) classified as diabetic patients with no DR and 28 (27.4%) classified as mild DR. The subjects included 42 men (41.2%) and 60 women (58.8%) with a mean age of 58.67 (±10.7). There was a significant difference in age (p = 0.015) and glycemia (0.015) between groups. The demographic values are shown in Table 1. There was no correlation between duration of DM, glycemia, glycosilated haemoglobin to GCL or RNFL or RT. Figures 1 and 2 show the influence of gender and age on two outcome software measures (GCL + IPL and RNFL). The female group showed a significant difference between groups in both measures (GCL + IPL, p = 0.004; RNFL, p = 0.001). The male group displayed a significant difference only in the GCL + IPL measure (p = 0.011). The age group less than 59 years showed a significant difference in RNFL (p = 0.002).

**Table 1:** **Demographics of the subjects in different groups**

**Patient demographics**				
**Parameter**	**Controls (N = 28)**	**DM with no DR (N = 46)**	**Mild DR (N = 28)**	***p***
Age, years*	54 ± 10	59 ± 10	62 ± 11	0.01
Gender, male:female	14:14	17:29	11:17	0.53
Glycemia, mg/dL*	92.50 ± 9.19	167.33 ± 134.44	148.81 ± 70.72	0.01
HbA1c, %	-	7.55 ± 1.57	7.16 ± 1.22	0.10
Duration of DM, years	-	12 ± 7	12 ± 9	0.98

The values are mean SD for all subjects in each group. “-”: not done. *Statistically significant difference between mild DR and controls (P <0.05).

A total of 102 sets of OCT images were obtained and analyzed. Thickness measurements of GCL + IPL and RNFL were significantly correlated with age (GCL, p = 0.001, R = −0.315; RNFL, p = 0.010, R = −0.254). There was no significant correlation between glycemia and the thicknesses, nor between HbA1c and thicknesses. However, the correlation between these clinical variables and thicknesses showed a negative Pearson coefficient (R), indicating that the higher the blood glucose or glycated hemoglobin, the smaller thicknesses would be.

### Automated analysis

**Figure 3:**
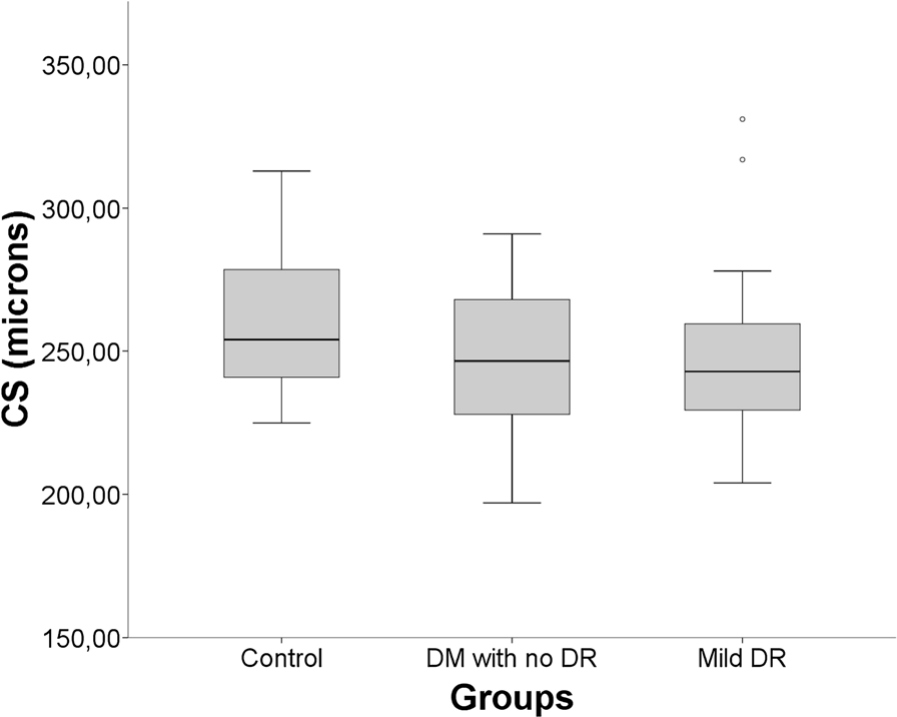
**Boxplot shows mean central subfoveal (CS) thickness of each study group measured with macular cube 200×200.** There was a slight reduction in diabetic groups compared to control but without statistical significance (P > 0.05).

**Figure 4:**
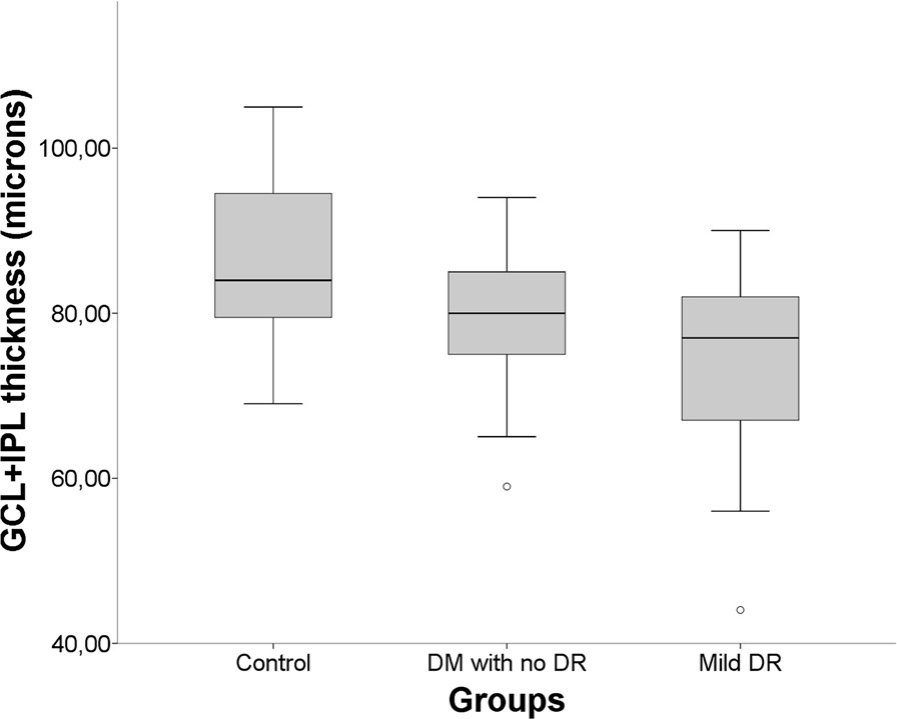
**Boxplot shows mean ganglion cell + inner plexiform layer (GC + IPL) thickness of each study group with macular cube 200×200.** Mean CGL + IPL and mean RNFL were thinner in the group with DM with no DR when compared to control (P < 0.05). ANOVA with Bonferroni post test indicated a statistically significant reduction in GCL + IPL in DM with no DR compared to control (P = 0.039).

**Figure 5:**
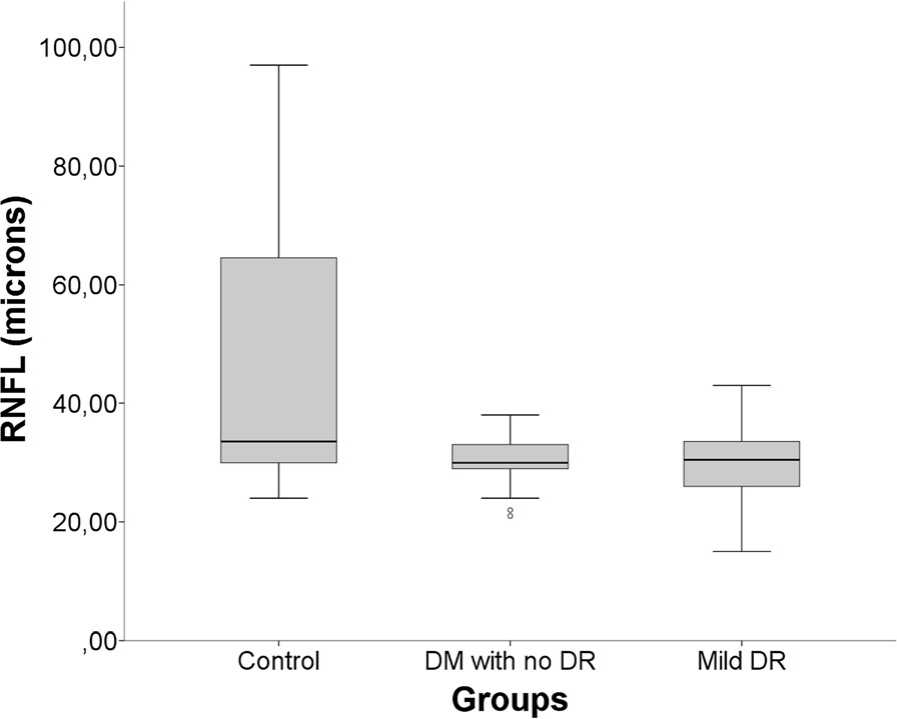
**Boxplot shows mean retinal nerve fiber layer (RNFL) thickness of each study group with macular cube 200×200.** There was a statistically significant reduction in RNFL in DM without DR or eyes with mild RD compared to the control group (P < 0.001).

In quantitative analysis with the Cirrus software, the GCL+ IPL and RNFL were thinner in the group with DM with no DR when compared to controls (p < 0.05). Furthermore, GCL+ IPL and RNFL were even thinner in patients with DR (Table 2). ANOVA with Bonferroni post test indicated a statistically significant reduction compared to controls (p < 0.05) in the following retinal layers: RT in mild DR group (p = 0.032); GCL + IPL in DM with no DR group (p = 0.039) and in mild DR group (p = 0.003); and RNFL in DM without DR or eyes with mild RD (p < 0.001) (Figures 3, 4 and 5).

**Table 2:** **Thickness measures by software analysis**

**Mean thickenss by software analysis (μm)**			
**Measure**	**Control (N = 28)**	**DM with no DR (N = 46)**	**Mild DR (N = 28)**
RT	284.07^a^ ± 13.40	279.02 ± 14.26	271.46^a^ ± 26.23
CS	260.61 ± 24.15	245.46 ± 24.36	254.68 ± 46.90
GCL + IPL	91.14^bc^ ± 32.89	79.78^b^ ± 7.36	73.96^c^ ± 10.61
RNFL	45.93^de^ ± 24.60	30.41^d^ ± 3.46	29.78^e^ ± 5.57

RT: Retinal thickness; CS: Central subfield of ETDRS grid; GCL + IPL: ganglion cell layer and inner plexiform layer; RNFL: Retinal nerve fiber layer.

The letters ^a, b, c, d, e^indicate significant difference between the groups (a: p = 0.032, b: p = 0.039,c: p = 0.003,d: p < 0.001 , e: p < 0.001).

A multiple linear regression model was used to assess the relationship between GCL thickness, RNFL thickness and RT with variables with correlation (age and DR status). In Table 3, standardized coefficients of the explanatory variables are presented. These variables show that DR status is the most explanatory variable to thickness reduction.

**Table 3:** **Standardized regression coefficients derived from multiple linear regression**

**Standardized regression coefficients derived from multiple linear regression**			
**Dependent variable:**	**Independent variable**	**Standartized coefficients**	***p***
RT (R = 0.293, p = 0.012)	Age	−0.154	0.126
	DR status	−0.210	0.038
RNFL (R = 0.427, p < 0.001)	Age	−0.156	0.103
	DR status	−0.358	<0.001
GCL (R = 0.406, p < 0.001)	Age	−0.252	0.010
	DR status	−0.256	0.009

Regression coefficients are presented in standardized (z-score) form.

### Sectored automated analysis

**Figure 6:**
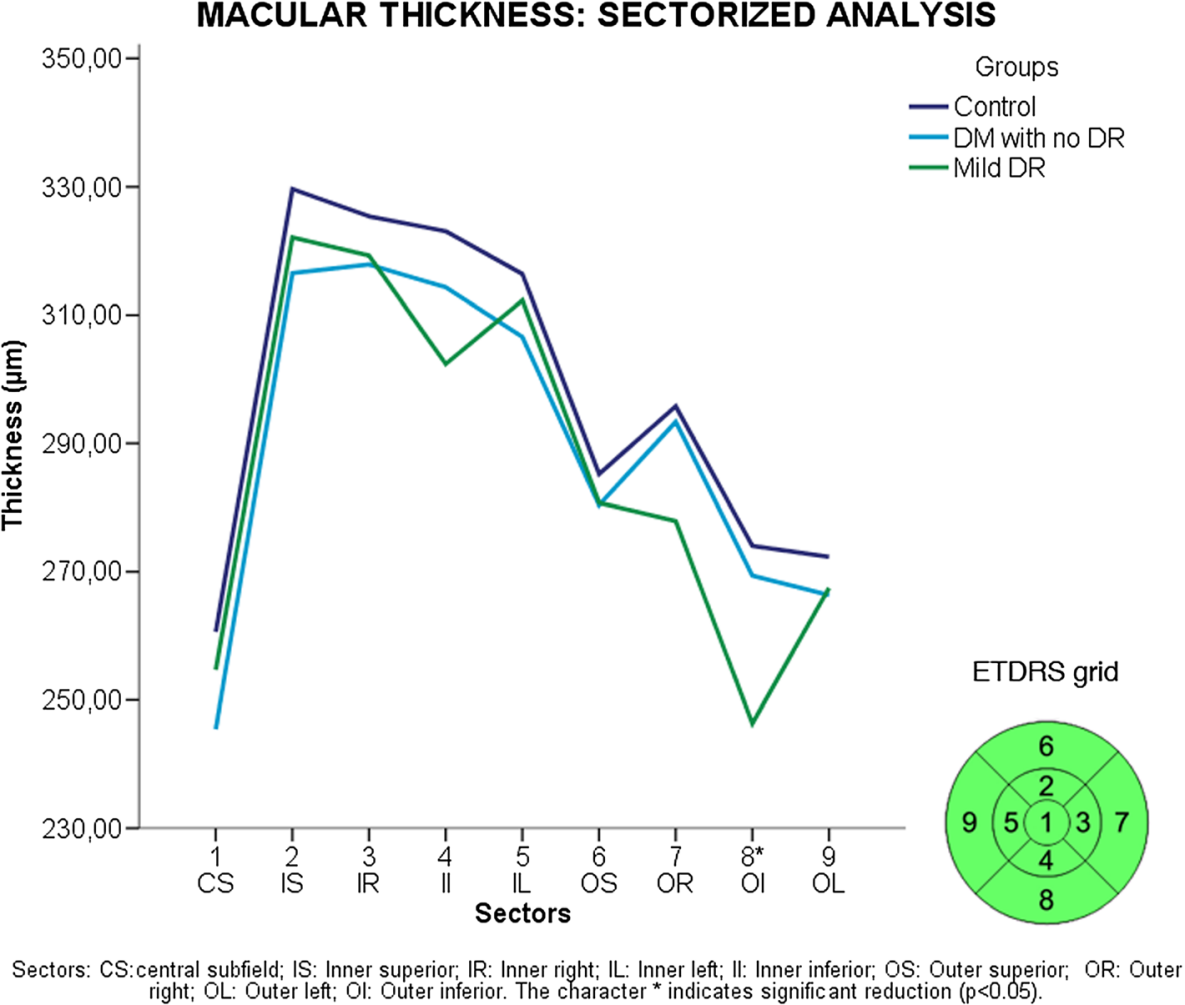
**Sectorized analysis of retinal thickness (RT) based on ETDRS macular grid.** Sectors: CS: central subfield; IS: Inner superior; IR: Inner right; IL: Inner left; II: Inner inferior; OS: Outer superior; OR: Outer right; OL: Outer left; OI: Outer inferior. The character *indicate significant reduction (p < 0.05).

**Figure 7:**
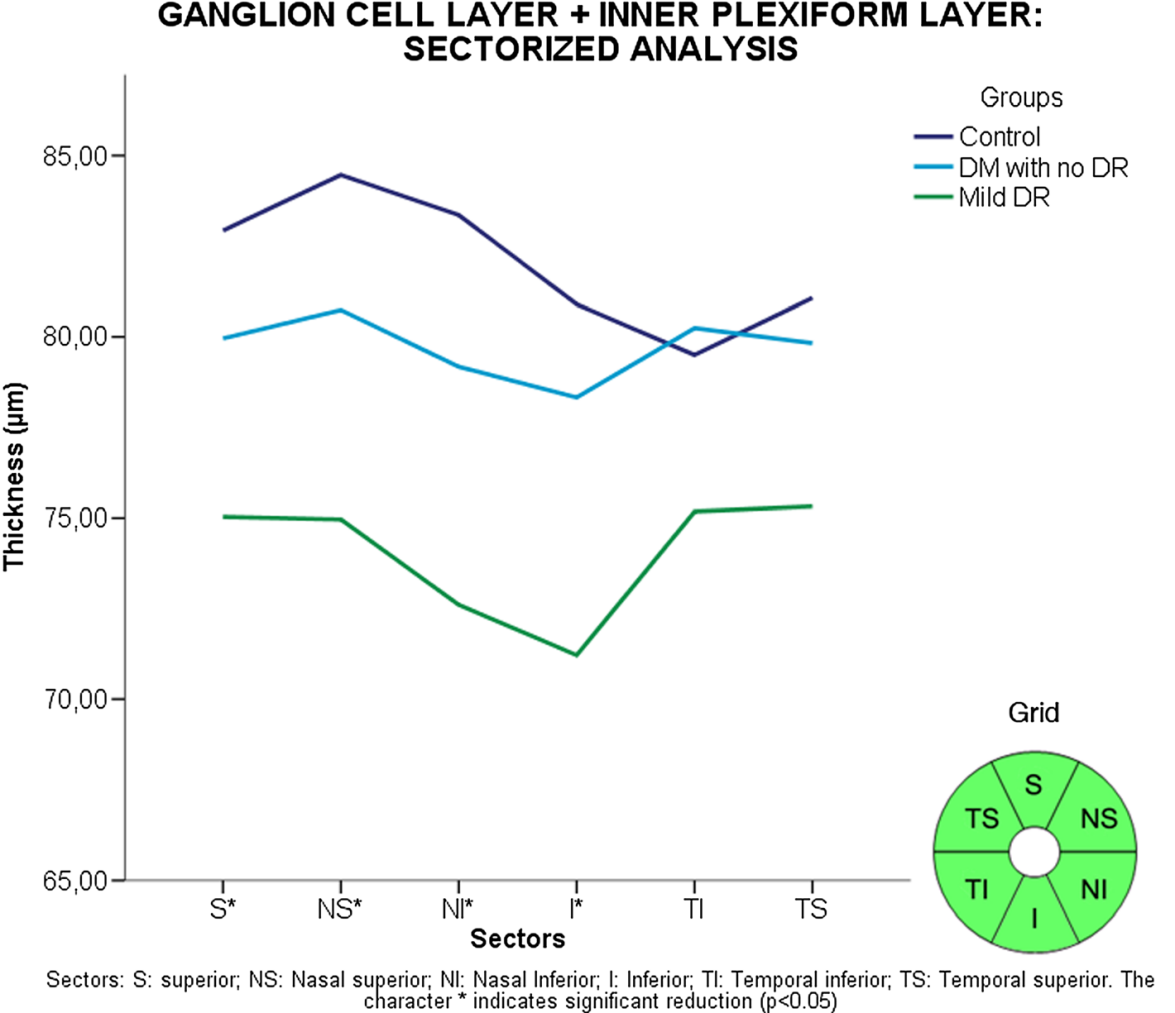
**Sectorized analysis of ganglion cell layer + inner plexiform layer (GCL + IPL) thickness.** Sectors: S: superior; NS: Nasal superior; NI: Nasal Inferior; I: Inferior; TI: Temporal inferior; TS: Temporal superior. The character * indicate significant reduction (p < 0.05).

**Figure 8:**
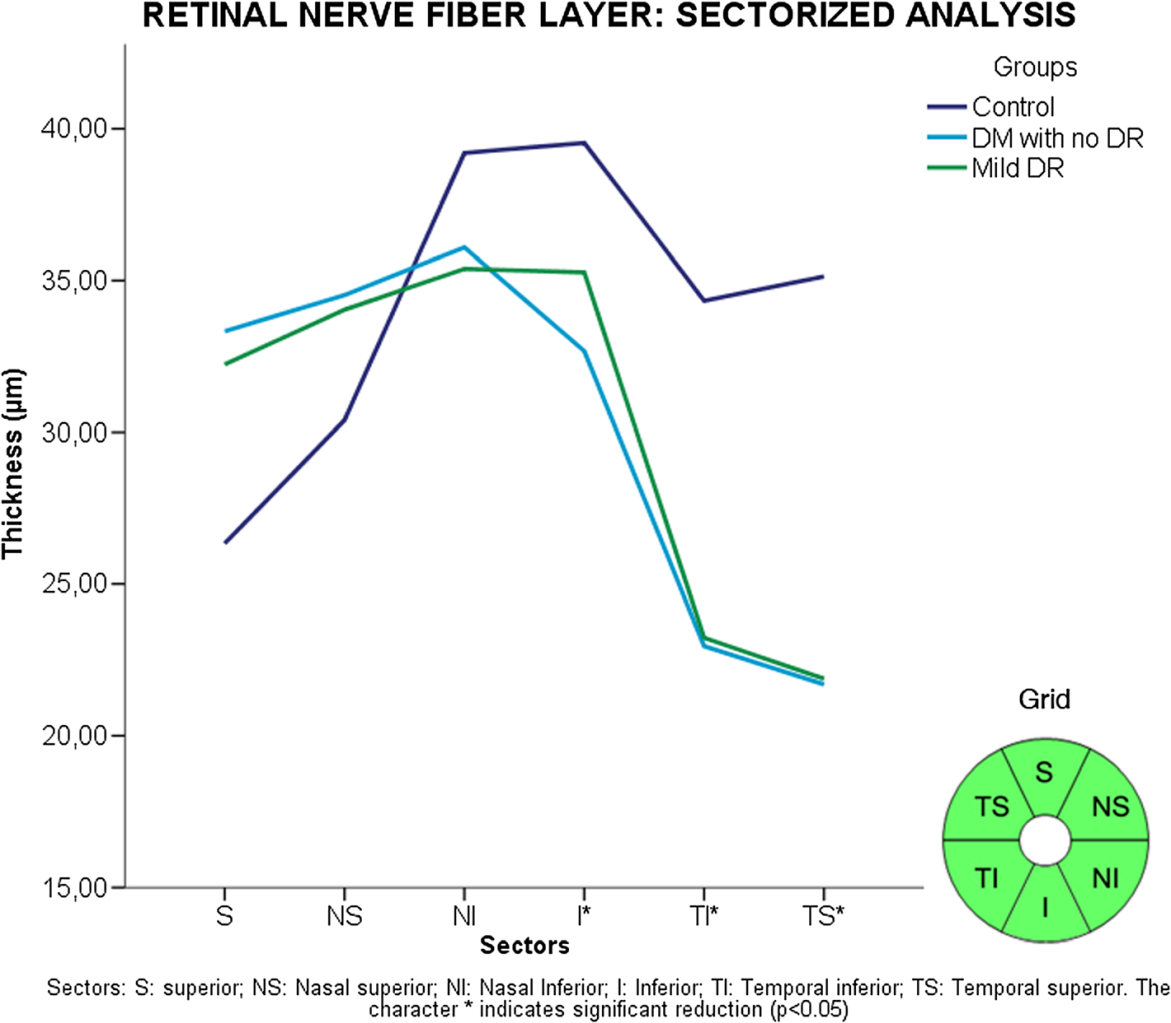
**Sectorized analysis of retinal nerve fiber layer (RNFL) thickness.** Sectors: S: superior; NS: Nasal superior; NI: Nasal Inferior; I: Inferior; TI: Temporal inferior; TS: Temporal superior. The character *indicate significant reduction (p < 0.05).

Using the ETDRS (Early Treatment Diabetic Retinopathy Study) grid proposed subdivision, significance was found in Macular thickness in the 8 grid (lower external) between the mild DR group and controls (P = 0.032) (Figure 6). Sectored analysis of GCL + IPL, there was a statistically significant difference between the mild DR group and controls in the following sectors: nasal inferior (P < 0.001), nasal superior (P = 0.002), inferior (P < 0.011) and superior (P = 0.034). In addition, significant differences in values were observed between DM without DR and mild DR groups in nasal inferior (P = 0.023) and inferior (P = 0.05) sectors (Figure 7). Sectored analysis of RNFL showed significant difference in the temporal superior sector between the DM without DR group and controls (P = 0.004) and between the mild RD group and controls (P = 0.01) (Figure 8).

### Subjective analysis

According to the subjective analysis of examiner 1, there was a significant difference between the groups in GCL, between the mild DR group and controls (P = 0.043), and in CS between the DM with no DR group and controls (P = 0.037). According to examiner 2, there were significant changes between the groups in GCL, between the DM with no DR group and controls (P < 0.001) and between the mild DR group and controls (P < 0.001). In analysis of reproducibility of measures between the two observers using intraclass correlation (ICC), good correlation in INL (0.77) and ONL (0.78) thickness measurements was achieved. The correlation between examiners in the measurements of GCL (0.64), IPL (0.52), OPL (0.69) and fovea (0.72) was considered satisfactory. RNFL measurement was considered have a poor correlation (0.171).

## Discussion

DR is the main cause of visual impairment and blindness in the adult working-age population, and it has been proposed to be primarily a retinal microvascular disorder [10]. However, several recent publications have shown that retinal neurodegeneration precedes clinically detectable microvascular damage [1,2,4,11,12]. The hypothesis referring to the occurrence of neurodegeneration before vascular damage has been confirmed by electrophysiological and psychophysical studies [13,14]. Recent studies in animals have revealed that diabetes causes the loss of different types of retinal cells including ganglion cells, bipolar cells, amacrine cells, horizontal cells, and eventually photoreceptors [3,4,14,15]. Clinically, different authors have reported a decrease in retinal thickness in diabetic eyes with or without clinical signs of DR compared to normal subjects [1,2,12,16-18]. Vujosevic and Midena as well as Van Dijk et al. have shown a reduction in the inner retinal thickness in the macula in diabetics with mild DR. Van Dijk et al. speculates an initial GCL loss in the pericentral areas followed by RNFL thinning in the peripheral macula [5]. In addition, Vujosevic and Midena found outer retina may not affect at early stages of DM and concluded that automatic intraretinal layering by SD-OCT may be a useful tool to diagnose and monitor early intraretinal changes in DR. In our study, we evaluated whether diabetic patients with no DR or mild DR had retinal changes measured by four parameters: GCL + IPL, RNFL, CS and RT.

The total macular thickness in diabetic eyes with no DR and mild DR was examined in our paper using CS and RT, and we found a significant reduction in eyes with mild DR in RT (P = 0.032). These findings matched the results of Biallosterski et al., who found that the mean RT in the pericentral area was decreased in patients with minimal DR compared to healthy controls. Verma et al. also found reduction in foveal thickness in patients with DM and no retinopathy compared to healthy individuals [6]. The reduction in RT later in patients with mild DR may mean that thinning in one or more retinal layers may indeed occur during disease evolution but requiring several years to appear.

RNFL evaluation with SD-OCT has been a reliable parameter in patients with glaucoma. Herein, analysis with Cirrus software disclosed reduction in RNFL both in patients with no DR and those with mild DR. Similar findings of decreased RNFL has been noticed by van Dijk et al. in patients with mild DR [1]. Such outcomes ought to be analyzed in conjunction with the results in the GCL + IPL, since RNFL death occurs as axonal loss of GCL. In our study, we demonstrated thinning of GCL + IPL both in patients with DM and no DR and those with mild DR. In addition, there was a statistically significant difference in GCL + IPL between the mild DR group and controls in the superior, nasal superior, nasal inferior, and inferior sectors. These results confirm previous studies both in animals and humans, showing GCL damage as a primary finding in patients with early DM [3,6,19-22]. Future studies should investigate the clinical significance of these outcomes and whether GCL may indeed be a particular cellular target of DM.

Some of strengths of our study included examination of both patients with DR or just DM and the objective analysis with the OCT software in addition to a subjective retinal measurement by two experimented examiners. On the other hand, a limitation of our investigation was the poor intraclass correlation between examiners in some retinal layers. Besides that, the difference in age between groups was another limitation. Despite this, a multiple linear regression model was used to assess the relationship between GCL thickness, RNFL thickness and RT with variables with correlation (age and DR status), which showed that DR status was the most explanatory variable. Other drawback of our investigation was the uncertain onset of the DM, as type 2 DM patients may have the disease for some time before they recognize the condition, but this fact would only underestimate the findings.

## Conclusions

Our paper reports early signs of neuroretinal changes by detecting a significant thinning of different inner cell layers and central retina in patients with early DR compared to normal eyes. The investigation encountered thinning in thickness of GCL and RFNL in patients with DM without DR, which suggests neuroretinal changes before vascular signs. Future studies should compare functional testing with electrophysiology and microperimetry with OCT findings.

## Abbreviations

DM: Diabetes mellitus
DR: Diabetic retinopathy
GCL: The ganglion cell layer + inner plexiform layer
RNFL thickness: Retinal nerve fiber layer
CS retinal thickness: Central subfoveal
RT thickness: Average macular thickness and total retinal
ANOVA: Analysis of Variance
OCT: Optical coherence tomography
HbA1c: Last glycosylated hemoglobin
FA: Fluorescein angiography
SD-OCT: Spectral-domain optical coherence tomography
ICC: Intraclass correlation coefficient
ETDRS: Early Treatment Diabetic Retinopathy Study
ICC: Intraclass correlation
